# ER Stress-Induced eIF2-alpha Phosphorylation Underlies Sensitivity of Striatal Neurons to Pathogenic Huntingtin

**DOI:** 10.1371/journal.pone.0090803

**Published:** 2014-03-03

**Authors:** Julia Leitman, Boaz Barak, Ron Benyair, Marina Shenkman, Uri Ashery, F. Ulrich Hartl, Gerardo Z. Lederkremer

**Affiliations:** 1 Department of Cell Research and Immunology, George Wise Faculty of Life Sciences, Tel Aviv University, Tel Aviv, Israel; 2 Sagol School of Neuroscience, Tel Aviv University, Tel Aviv, Israel; 3 Department of Neurobiology, George Wise Faculty of Life Sciences, Tel Aviv University, Tel Aviv, Israel; 4 Department of Cellular Biochemistry, Max Planck Institute of Biochemistry, Martinsried, Germany.; Boston University School of Medicine, United States of America

## Abstract

A hallmark of Huntington’s disease is the pronounced sensitivity of striatal neurons to polyglutamine-expanded huntingtin expression. Here we show that cultured striatal cells and murine brain striatum have remarkably low levels of phosphorylation of translation initiation factor eIF2α, a stress-induced process that interferes with general protein synthesis and also induces differential translation of pro-apoptotic factors. EIF2α phosphorylation was elevated in a striatal cell line stably expressing pathogenic huntingtin, as well as in brain sections of Huntington’s disease model mice. Pathogenic huntingtin caused endoplasmic reticulum (ER) stress and increased eIF2α phosphorylation by increasing the activity of PKR-like ER-localized eIF2α kinase (PERK). Importantly, striatal neurons exhibited special sensitivity to ER stress-inducing agents, which was potentiated by pathogenic huntingtin. We could strongly reduce huntingtin toxicity by inhibiting PERK. Therefore, alteration of protein homeostasis and eIF2α phosphorylation status by pathogenic huntingtin appears to be an important cause of striatal cell death. A dephosphorylated state of eIF2α has been linked to cognition, which suggests that the effect of pathogenic huntingtin might also be a source of the early cognitive impairment seen in patients.

## Introduction

A so far unexplained phenomenon in many neurodegenerative diseases is the high sensitivity of certain specific cell types of the central nervous system. This is also true in Huntington’s disease (HD), which initially affects medium spiny neurons in the brain striatum [1], [2], and only later regions of the brain cortex. The reasons for the special sensitivity of striatal cells are unknown, though mechanisms have been proposed involving proteins with enhanced expression in these cells [3]. HD is a progressive, fatal genetic disorder affecting cognition and movement, which arises from mutant forms of the huntingtin (Htt) protein with expanded polyglutamine (polyQ) tracts (>35 amino acids). This mutation causes Htt aggregation, which interferes with normal cell metabolism [4], [5], [6], leading to cytotoxicity through a yet unclear mechanism. One of the effects of the expression of mutant Htt is the activation of the unfolded protein response (UPR) [7], [8], [9], [10], and an influence on autophagy [11], [12], reviewed in [13], [14]. UPR activation occurs by interference with the ubiquitin-proteasome system (UPS) [15], [16], [17] and ER-associated protein degradation (ERAD) [8], [18], a pathway that reduces the protein load in the ER [19]. This interference leads to an overload of unfolded or misfolded proteins in the ER, termed ER stress, which triggers the UPR. In mammals, the UPR includes three signaling pathways, initiated by their sensors, the ER-resident transmembrane proteins PERK, activating transcription factor-6 (ATF6), and inositol-requiring enzyme-1 (IRE1) [20]. Here we investigated whether there are differences in early and late markers of the UPR branches in response to ER stressors and to pathogenic huntingtin expression in stable murine striatal cell lines expressing a full-length wild type (WT) Htt form (ST*Hdh* ^Q7/7^) or a polyQ-expanded Htt (ST*Hdh* ^Q111/111^) compared to other cell types. A general accumulation of glycoproteins in the ER had been reported in ST*Hdh* ^Q111/111^ cells [21]. Here we show that there is a significant difference in the PERK branch of the UPR in the striatal neurons. Upon activation, by its sensing of ER stress, PERK undergoes autophosphorylation and phosphorylates eIF2α, inactivating eIF2 and inhibiting general protein synthesis. Striatal cells showed a much lower level of eIF2α phosphorylation than other cell types, which was much increased by pathogenic Htt expression. This was also true when analyzing the striatum compared to other regions in murine brain sections. We exploited this phenotype of striatal neurons by inhibiting the PERK pathway, compensating for the toxic effect of polyQ-expanded Htt.

## Materials and Methods

### Materials

Promix cell labeling mix ([^35^S]Met plus [^35^S]Cys), >1000 Ci/mmol was from PerkinElmer (Boston, MA). MG-132, tunicamycin (Tun), PKR inhibitor (PKRi), poly-I:C, DMEM Cys/Met-free, Trichloroacetic acid (TCA) and other common reagents were from Sigma. Guanabenz (Gz) was a kind gift of Anne Bertolotti. PERK inhibitor A4 [22] was from ChemBridge Chemical Store.

### Plasmids and constructs

The eIF2αGFP expressing vector was described before [23].

### Antibodies

Rabbit antibodies were as follows; anti-BiP from Sigma or a kind gift of Linda M. Hendershot, anti-phospho-eIF2α (Ser51), anti-phospho-PERK and anti-DARPP-32 (a kind gift of Beth Stevens) from Cell Signaling, anti-GFP from Santa Cruz, anti-CHOP and anti-GADD34 were a kind gift of David Ron. Mouse monoclonal antibodies were as follows; anti-GAPDH from Chemicon International, anti-total eIF2α from Cell Signaling and anti-ATF6 from Abnova. Goat anti-mouse IgG conjugated to Cy2 or to dylight 549, goat anti-rabbit IgG conjugated to Cy3 or to dylight 488, and goat anti-rabbit and anti-mouse IgG conjugated to HRP were from Jackson Labs. Normal goat serum was from Vector Laboratories (Burlingame, CA).

### Cell culture and transfections

HEK 293T, NIH 3T3 and N2a cells were grown in DMEM plus 10% FCS at 37°C under 5% CO_2_. ST*Hdh* ^Q7/7^ and ST*Hdh* ^Q111/111^ cells [21] were a kind gift of Marcy E. MacDonald and grown as described previously [21]. HEK 293T cells were transfected according to the calcium phosphate method.

### Treatments and immunoblotting

Cells were treated with 5 µg/ml or 10 µg/ml Tun or with 15 µM or 40 µM MG-132. Gz, A4, PKRi and poly-I:C were added to the cell medium at the indicated concentrations and times. The concentration range was chosen in accordance to previous reports [22], [24], [25]. Cells were lysed with PBS containing 1% Triton X-100, 0.5% Sodium deoxycholate with protease inhibitors and centrifuged to pellet debris and nuclei. 10 mM sodium fluoride (NaF) and 10 mM β-glycerolphosphate (βGP) were added to the lysis buffer for detection of phosphorylated proteins. The supernatants were separated from pellets and boiled in loading buffer for 5 min. Immunoblotting, detection by ECL and quantitations were done as described previously [26].

### RT-PCR

Total cell RNA was extracted using TRIzol reagent (Invitrogen), reverse transcription and PCR were described previously [26]. Primers for *Ppp1r15b/CreP* amplification were AGGCTCCTTTTCAACCGTCAGGG and CCAGGAAGGTTACCTTTTTTCTCTTG. Primers for spliced XBP1 amplification were TCTGCTGAGTCCGCAGCAG and GAAAAGGGAGGCTGGTAAGGAAC and for GAPDH amplification, TGGCCTCCAAGGAGTAAGAA and GGAAATTGTGAGGGAGATGC.

### *In vitro* dephosphorylation assay

HEK 293T cells were transfected with an eIF2αGFP-expressing vector, grown for 2 days and treated with Tun (10 µg/ml) for 2 h to obtain high levels of phosphorylated eIF2αGFP. Cell lysate (1% NP40 with protease inhibitors) served as a substrate for eIF2αGFP-P dephosphorylation. NIH 3T3, N2a, STHdh^Q7/7^ and STHdh^Q111/111^ cells grown in parallel were lysed in the same conditions. The same amounts of protein from each cell line were mixed with a constant amount of the substrate and incubated at 37°C for 4 h or immunoblotted separately for the detection of the total input. The substrate mixed with lysis buffer served as a control and was incubated in parallel either at 37°C or at 4°C. All the samples were then boiled with sample buffer and run on 10% SDS-PAGE. The signal of eIF2αGFP-P in each lane, detected with anti-eIF2α-P, was normalized to the total protein input of each cell line and to the total eIF2αGFP detected with anti-GFP antibody.

### Immunofluorescence

Cells grown on coverslips in 24 well plates were fixed with 3% paraformaldehyde, followed by permeabilization with 0.5% triton X-100 in PBS and blocking with 50 mM glycine in PBS and normal goat IgG in PBS/ 2% BSA. The cells were incubated with primary antibodies for 1 hour, washed and incubated for 30 minutes with secondary antibodies, followed by washes. Nuclei were stained with DAPI. The samples were and observed using a Zeiss laser scanning confocal microscope (LSM 510 Meta; Carl Zeiss, Jena, Germany). The acquired images were analyzed in ImageJ.

### Total protein synthesis measurements

For estimation of general translation rates cells were labeled for 20 min with [^35^S] Met + Cys (20 µCi/ml), followed by three washes with PBS. Cell lysis was performed with 1% Triton X-100 in PBS and protease inhibitors. Triplicate samples of cell lysates containing 20 µg of total protein were applied onto Whatman 3 MM filters and boiled three times for 1 min with 5% trichloroacetic acid containing excess of unlabeled Met + Cys. Filters were rinsed in ethanol, dried and analyzed in a scintillation counter (Beckman).

### Preparation of brain sections

Brains from transgenic male N171-82Q mice expressing an N-terminal fragment of Htt (first 171 amino acids) with 82 glutamines (N = 6) [27] (Jackson laboratories) and their WT littermates (N = 10), all 20–22 weeks old were a kind gift of M. Mattson, M. Mughal and H. van Praag [28]. All procedures using these mice were approved by the institutional Animal Care and Use Committee of the National Institute on Aging (USA). Mice were anesthetized with ketamine and xylazine and sacrificed by transcardiac perfusion with 0.9% saline followed by perfusion with 4% paraformaldehyde (PFA) in 0.1 M phosphate buffer, pH 7.4. Brains were removed and fixed in 4% PFA in 0.1 M phosphate buffer overnight at 4°C, and then left in 30% sucrose for 2 nights at 4°C. Brains were cut on a freezing microtome at the level of the frontal cortex and cerebellum into a series of eight adjacent 30-µm thick coronal sections and collected into a cryoprotectant solution (30% ethylene glycol, 30% glycerol) in PBS, pH 7.4 and stored at –20°C until use.

### Staining of brain sections

Free-floating sections were washed with PBS to remove any remnants of cryoprotectant solution. Blocking was done with 20% normal goat serum in 1% Triton X-100/PBS (PBST) for 4h at room temperature. Primary antibodies were diluted in 2% goat serum/PBST. The slices were incubated with primary antibodies (rabbit anti-eIF2α-P, mouse anti-total eIF2α, rabbit anti-DARPP-32) for 30 min at 37°C, followed by incubation over 2 nights at 4°C. After rinsing with PBST, the sections were incubated with goat anti-rabbit IgG-Cy3 and goat anti-mouse IgG-Cy2 in 2% goat serum/PBST for 1 h, rinsed with PBST and incubated with DAPI (4',6-diamidino-2-phenylindole) for 5 min, followed by a final rinse and mounting. To minimize variability, sections from all animals were stained and treated simultaneously. Control rabbit and mouse antibodies were used instead of the specific primary antibodies to evaluate background staining. The slides were kept at 4°C in the dark and images were acquired using an LSM 510 Meta confocal microscope.

The images were analyzed with ImageJ with CellInt macro, kindly provided by Dr. Doron Kaplan (Tel Aviv University). Fluorescence intensity of eIF2α-P staining was normalized to cell number, which was counted according to total eIF2α staining, independently of its intensity. Threshold for cell counting was according to the Otsu algorithm and for background according to the Moments algorithm for all the images. EIF2α-P levels in brain regions relative to cortex were calculated for each section and then averaged; HD cortex eIF2α-P level was corrected according to the difference in the average of fluorescence intensity of eIF2α-P staining between HD and WT sections.

### Cell Cycle FACS analysis

Cells were treated as indicated, collected, washed with PBS, fixed with cold methanol and stored at –20°C. Before the FACS analysis cells were washed with PBS, propidium iodide (PI) solution (10 µg/ml) was added and the samples were read by flow cytometry. Cells in sub-G0/G1 were counted as apoptotic/ dead cells.

### Statistical Analysis

Data are expressed as means ± SE. Student’s t-test (unpaired, two -tailed) was used to compare the two groups, and the P value was calculated in GraphPad Prism 5 (GraphPad Software). P<0.05 was considered as statistically significant.

## Results

### Striatal neurons show a different response to ER stressors than other cell types. The response is affected by pathogenic huntingtin expression

**Figure 1:**
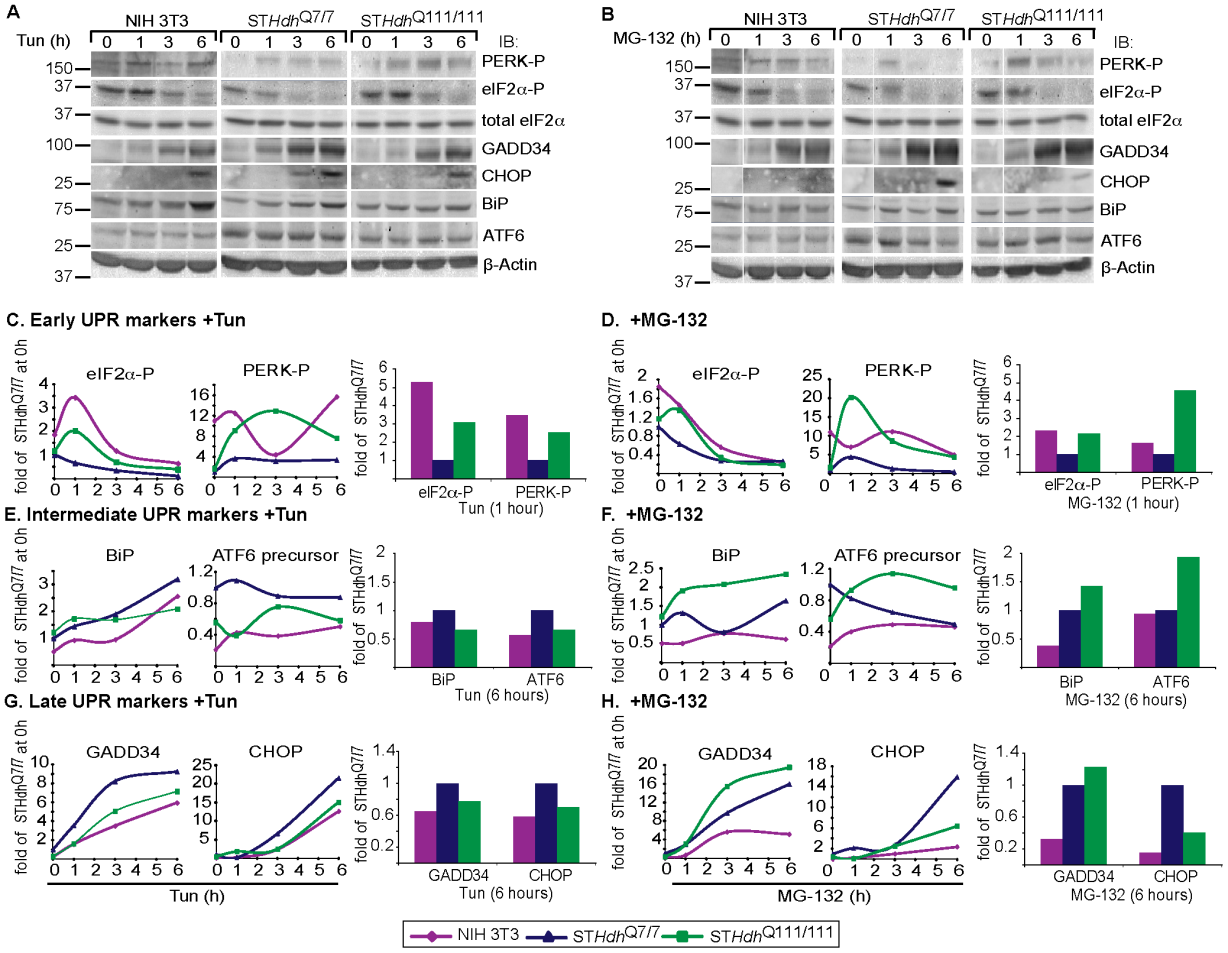
Striatal neurons show a low induction of early UPR markers, whereas later ER stress responses are upregulated. Early responses and in some cases late ones are increased by expression of Htt111Q. A,B) Levels of UPR markers after short term ER stress. ST*Hdh* ^Q7/7^ were compared to ST*Hdh* ^Q111/111^ and NIH 3T3 cells. Immunoblots show results of a representative experiment of 3. Vertical lines indicate removal of irrelevant lanes. C-H) Quantification of A,B. ST*Hdh* ^Q7/7^ cells do not activate properly an early stress response, mediated by PERK-P and its target eIF2α-P, induced by Tun (C, 10 µg/ml) or by MG-132 (D, 40 µM). In ST*Hdh* ^Q111/111^ cells, PERK-P and eIF2α-P are induced (C,D). Later ER stress responses are increased in ST*Hdh* ^Q7/7^ compared to NIH 3T3 cells (E-H). Htt111Q expression causes even more enhanced upregulation of the UPR markers in some cases. Values were normalized to β-actin levels as a loading control.

Given that mutant Htt induces ER stress [7], [8], [9], [10], we investigated how striatal cells handle the stress compared to other cell types. We compared murine NIH 3T3 fibroblasts with stable murine striatal cell lines with knock-in of a full-length Htt form with a polyQ stretch in the wild type (WT) range (7Q) or with a polyQ-expanded Htt (111Q) (ST*Hdh* ^Q7/7^ and ST*Hdh* ^Q111/111^ respectively) [21]. We treated cells with two different chemicals, an inhibitor of N-glycosylation, tunicamycin (Tun), which causes accumulation of misfolded unglycosylated proteins and a proteasomal inhibitor (MG-132), which prevents the degradation, causing accumulation of both secretory and non-secretory proteins. Following 1h of Tun or MG-132 treatments, early UPR activation, as revealed by the phosphorylated levels of the translation initiation factor eIF2α and of its kinase PERK, was unusually low in ST*Hdh* ^Q7/7^ and increased in ST*Hdh* ^Q111/111^ cells, comparable to the levels induced in murine NIH 3T3 fibroblasts (Fig. 1A-D). However, ST*Hdh* ^Q7/7^ cells showed a similar induction of the IRE1 branch of the UPR to NIH 3T3 cells, as measured by the levels of XBP1s mRNA. XBP1s induction was slower in ST*Hdh* ^Q111/111^ cells (Fig. S1). After longer exposure to ER stressors, ST*Hdh* ^Q7/7^ and ST*Hdh* ^Q111/111^ cells showed an increased induction of intermediate (BiP and ATF6) and late (pro-apoptotic) UPR markers, GADD34 and C/EBP homologous protein (CHOP) as compared to NIH 3T3 cells (Fig. 1E-H).

### Striatal cells have very low phosphorylation of eIF2α compared to other cell types, which is increased by expression of pathogenic huntingtin

**Figure 2:**
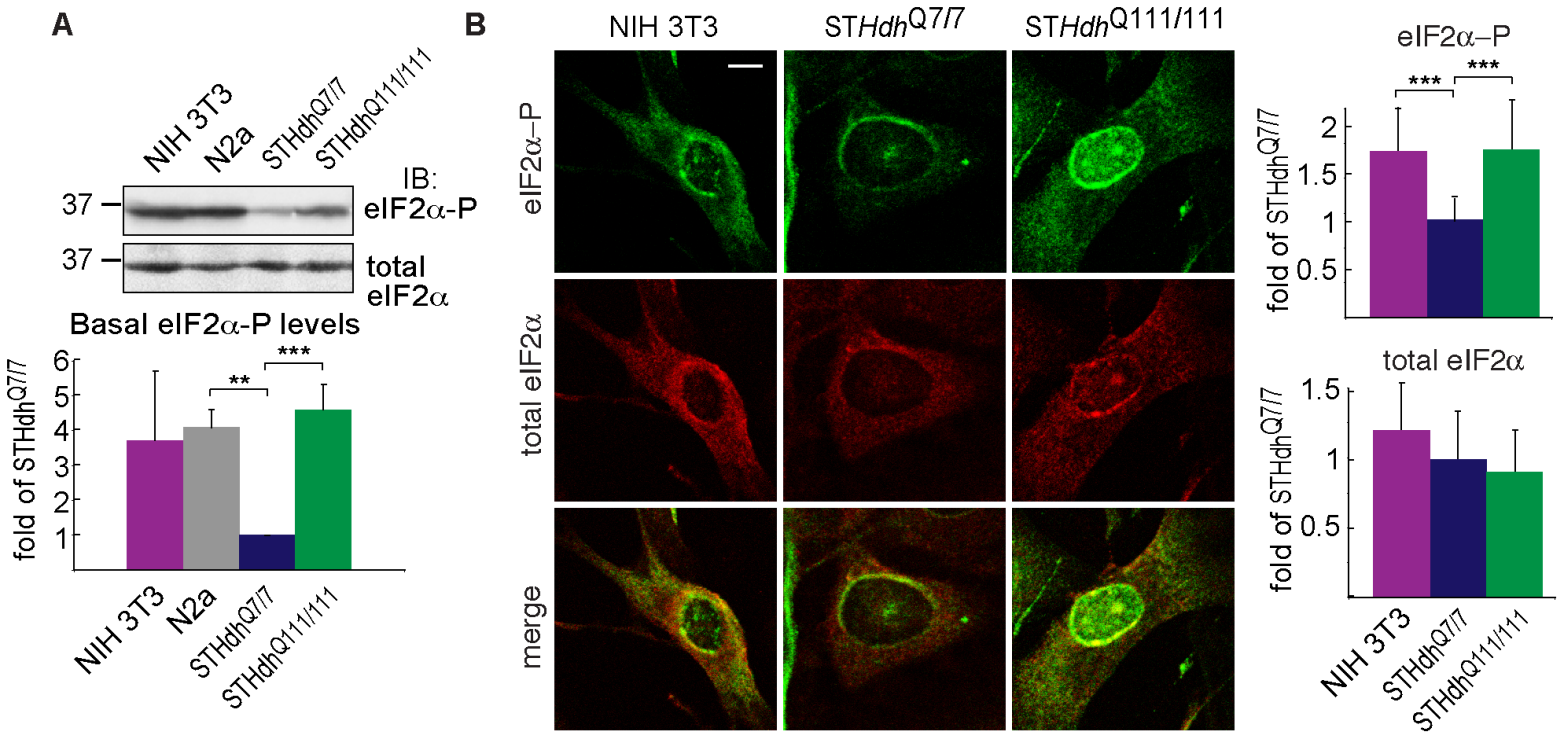
Very low eIF2α-P levels in striatal cells, much increased by expression of Htt111Q. **A**) Basal level of eIF2α-P in murine cell lines normalized by total eIF2α. Graph: average of 3 experiments ± SE**.** **P = 0.004, ***P  = 0.001. **B**) Immunofluorescence images of cells fixed, permeabilized and stained with rabbit anti-eIF2α-P and mouse anti-eIF2α followed by secondary antibodies. Bar = 10 µm. Image exposure time was kept constant to be able to compare protein levels in the different cell types. Levels relative to ST*Hdh* ^Q7/7^ levels were quantified from images from 3 experiments ± SE (>20 cells, ***P<0.001).

It was surprising to find that the basal levels of eIF2α-P were extremely low in the ST*Hdh* ^Q7/7^ cells compared to other murine cell lines, NIH 3T3 fibroblasts and N2a neuroblastoma cells (Fig. 2A). Expression of Htt111Q increased eIF2α-P in the striatal neurons to the basal levels that existed in the other cell types (Fig. 2A). Very low eIF2α-P levels in ST*Hdh* ^Q7/7^ cells and increase in ST*Hdh* ^Q111/111^ cells could also be observed by immunofluorescence (Fig. 2B). Interestingly, most of the increased eIF2α-P was located in the nucleus in ST*Hdh* ^Q111/111^ cells.

**Figure 3:**
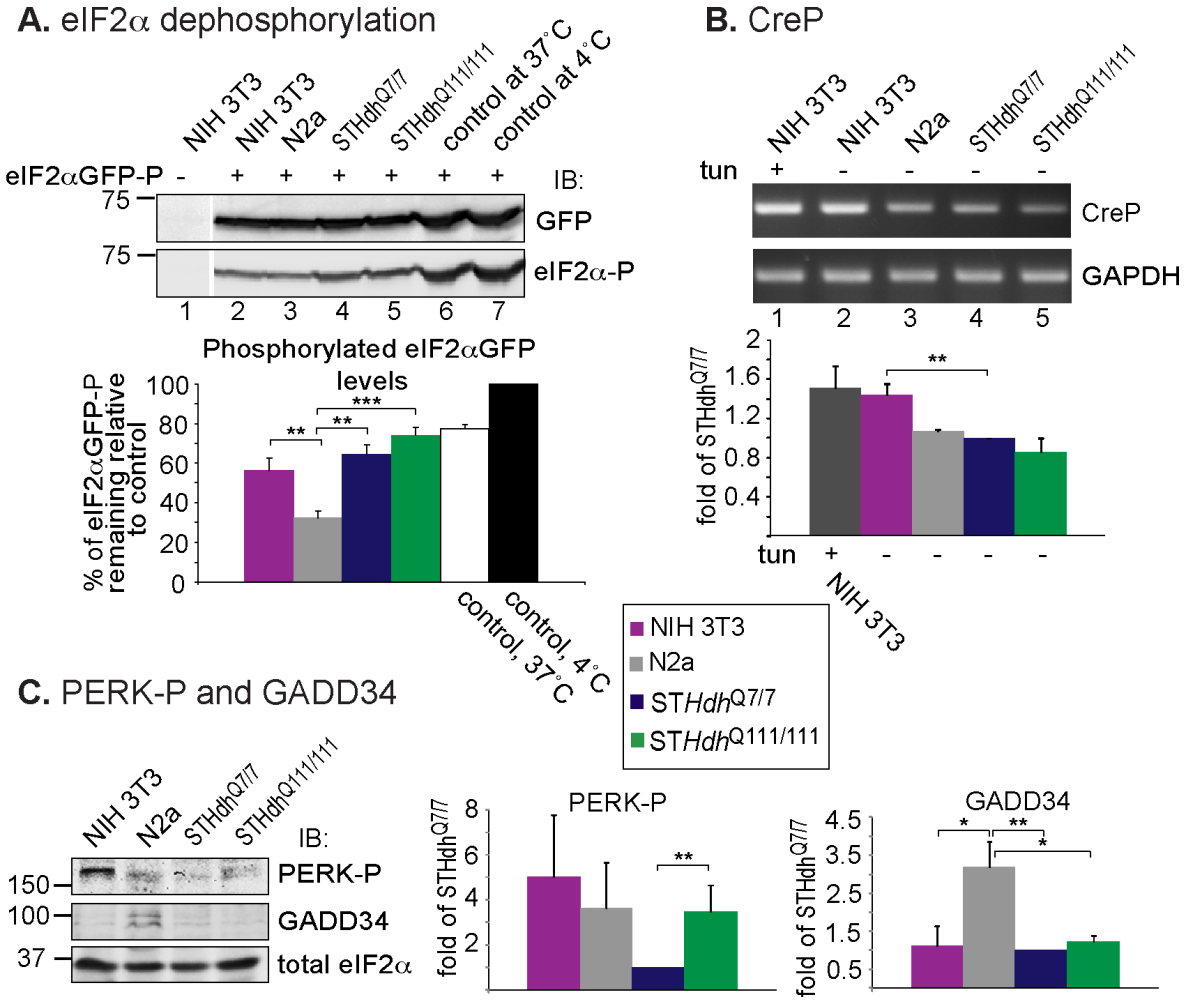
Low eIF2α-P levels in striatal cells are due to reduced phosphorylation (PERK activity), not increased de-phosphorylation. A) Striatal cells have very low eIF2α-P dephosphorylating activity, measured *in vitro* at 37°C with eIF2αGFP-P (Materials and Methods). **P  = 0.01, ***P = 0.001. B) Striatal cells have a very low basal level of CreP (RT-PCR of CreP mRNA). NIH 3T3 cells treated overnight with Tun, compared to the untreated cells, served as a control of UPR-independent constitutive CreP expression. **P  = 0.01. C) Striatal cells have a very low basal level of PERK-P, which is increased in ST*Hdh* ^Q111/111^ cells, and a low basal level of GADD34. *P <0.04, ** P (PERK-P) = 0.01, P (GADD34) = 0.002.

To explore whether the low striatal cell levels of eIF2α-P were the result of low phosphorylation or of a very active dephosphorylation, we incubated lysates from the different cell types with phosphorylated eIF2αGFP (eIF2αGFP-P) [23] *in vitro* at 37°C. The striatal cell lysates had actually a very low dephosphorylating activity, suggesting that their low levels of endogenous eIF2α-P are due to reduced phosphorylation levels and not increased dephosphorylation (Fig. 3A). Consistently, the basal levels of the regulatory subunits of protein phosphatase 1 (PP1), GADD34 (UPR-induced) and CReP (constitutive) were relatively low in the striatal cell lines (Fig. 3B,C). In line with what we observed for eIF2α-P, the level of the eIF2α kinase, PERK-P (the active form), was very low in the WT ST*Hdh* ^Q7/7^ cell line and increased upon expression of Htt111Q (Fig. 3C).

### Mouse brain striatum also presents a low level of eIF2α-P, which is increased by pathogenic huntingtin

**Figure 4:**
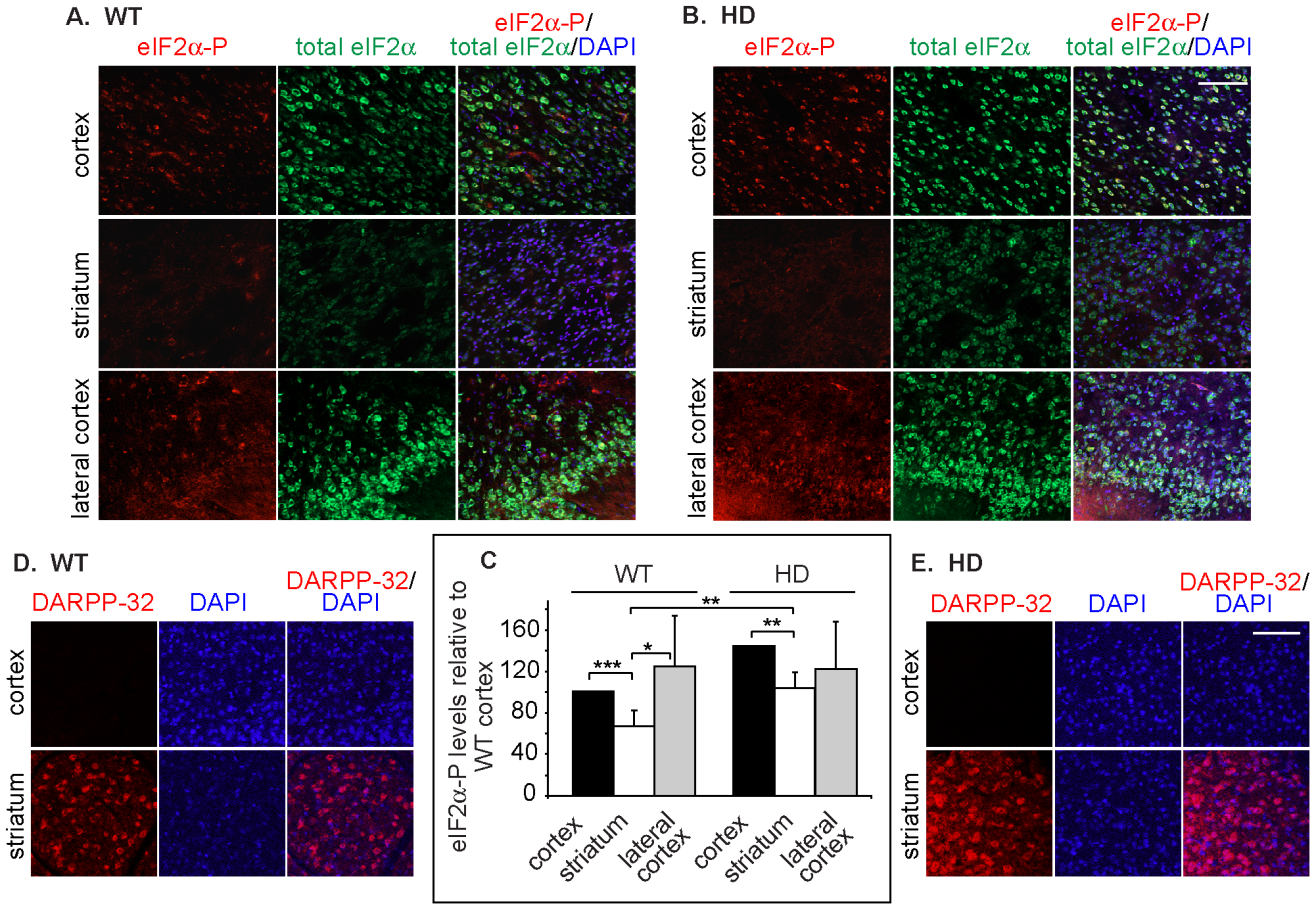
Mouse brain striatum presents a low level of eIF2α-P compared to other brain areas, which is increased in an HD mouse model. A-C) EIF2α-P levels in the striatum of WT mouse brains are significantly lower than those in other brain regions (A, N = 10) and are increased in HD model mouse (N171-82Q) brains (B, N = 6) compared to WT. Bar = 100 µm. Levels were quantified as explained in Materials and Methods (C). *P = 0.015, **P<0.003, ***P = 0.0001. D-E) The striatum marker DARPP-32 was stained in brain sections of the same WT (D) and HD model mice (E), labeling the striatum and not the cortex.

To investigate whether the low eIF2α-P levels in ST*Hdh* ^Q7/7^ cells and the increase in ST*Hdh* ^Q111/111^ are a phenomenon related to these cell lines or if it reflects the physiology of the mouse brain, we analyzed brain coronal sections from model HD transgenic mice expressing Htt171-82Q [27], compared with their WT littermates. Similarly to what we observed in the cell line, the striatum in the WT mice showed very low levels of eIF2α-P compared to other brain regions, the cortex and lateral cortex (Fig. 4A,C). EIF2α-P levels were higher in the Htt171-82Q mice compared to WT mice, but still relatively lower in the striatum than in other brain regions (Fig. 4B,C). Interestingly, also the levels of total eIF2α were unusually low in the striatum. In contrast, the striatum marker DARPP-32 strongly labeled the striatum of the Htt171-82Q mice and WT mice and not the cortex (Fig. 4D,E).

### Toxicity of pathogenic huntingtin in striatal neurons can be reversed by PERK inhibition

**Figure 5:**
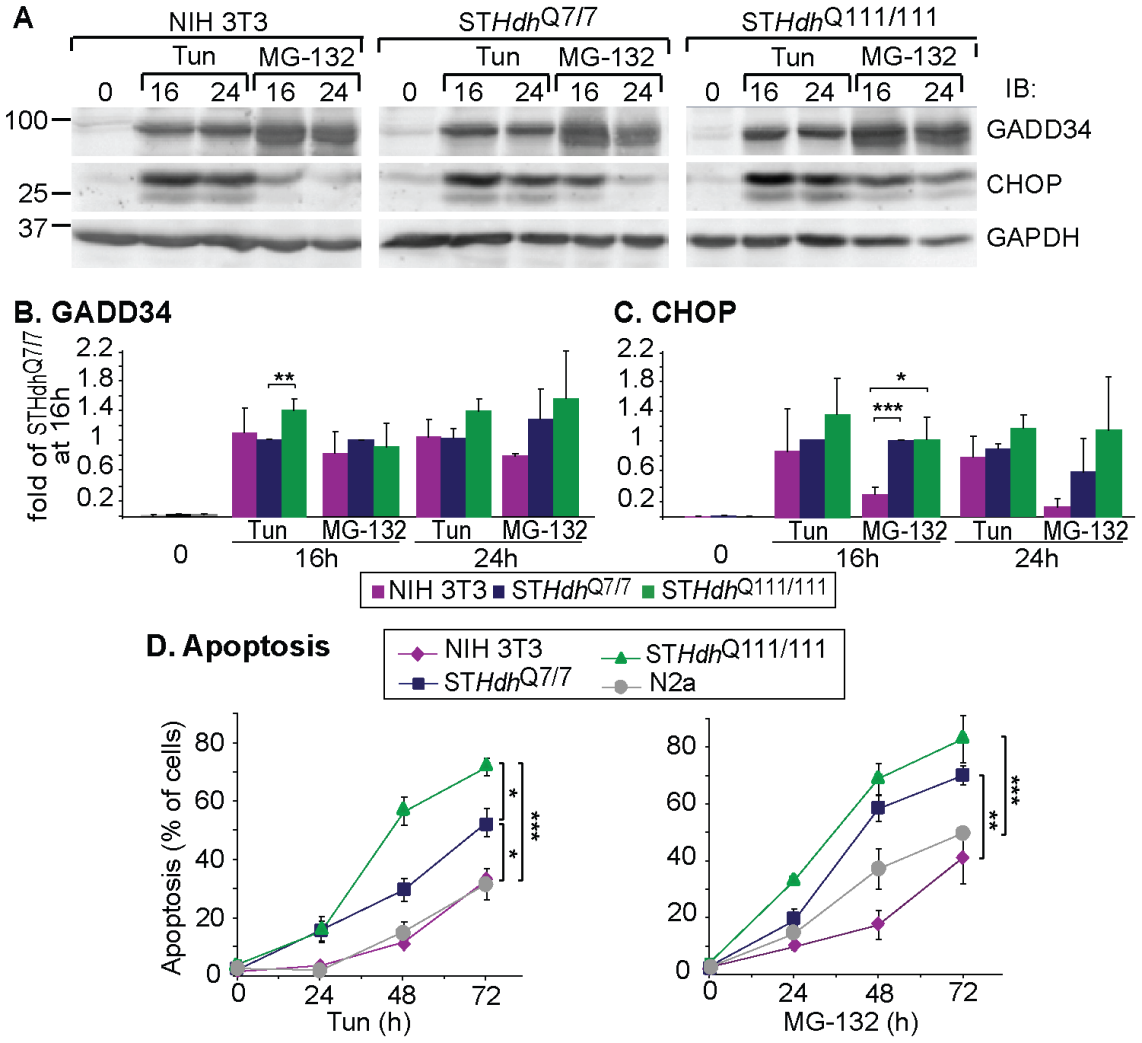
High sensitivity of striatal neurons to ER stress, further aggravated by expression of pathogenic huntingtin. **A-C**) Strong induction of GADD34 and CHOP upon prolonged ER stress in ST*Hdh* ^Q7/7^ cells and even stronger in ST*Hdh* ^Q111/111^ cells; (3 independent experiments ±SE). *P = 0.02, **P = 0.01, ***P = 0.0002. Immunoblots of a representative experiment are shown in A. GAPDH levels served here as a loading control. **D**) Prolonged ER stress induced with Tun or MG-132 leads to extensive death of ST*Hdh* ^Q7/7^ cells, further aggravated in ST*Hdh* ^Q111/111^ cells, as measured by FACS analysis of cell cycle progression with propidium iodide (PI) (see Fig. S2); (6 independent experiments ± SE). *P<0.05, **P = 0.01, ***P = 0.001.

We looked at the effect of prolonged treatments of the striatal cells with Tun and MG-132 to analyze their sensitivity compared to other cell types. Extended treatments (16-24h) with Tun or MG-132 led to the induction of the pro-apoptotic GADD34 and CHOP in all cell types, but more pronouncedly in ST*Hdh* ^Q111/111^ cells (Fig. 5A-C). When analyzing the rates of cell death, the striatal cells were much more sensitive than NIH 3T3 and N2a cells and showed much increased apoptosis after 48–72 h of treatment (Fig. 5D and Fig. S2). Expression of Htt111 further increased the death rate of the striatal cells, consistent with previous reports that used this and other models [8], [29]. Despite the toxicity of Htt111Q, late UPR markers and cell death were low in untreated ST*Hdh* ^Q111/111^ cells, as it is a cell line that has adapted to the basal stress [21].

**Figure 6:**
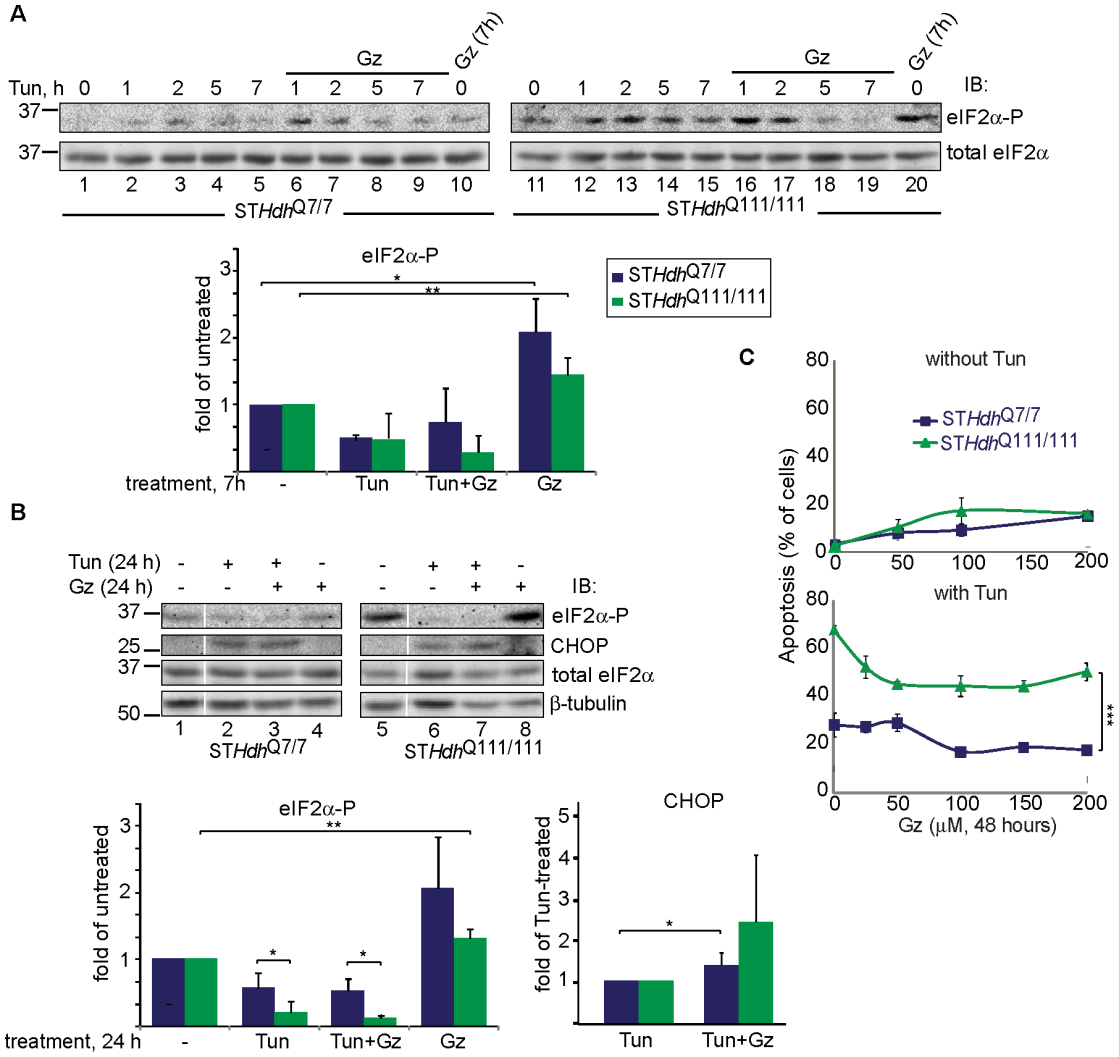
Regulation of phosphorylated eIF2α levels by inhibition of its dephosphorylation. **A**) Guanabenz (Gz), at a relatively high concentration (100 µM), inhibits eIF2α dephosphorylation in untreated ST*Hdh* ^Q7/7^ cells and also in those treated with Tun (5 µg/ml) up to 7h; this is also true in ST*Hdh* ^Q111/111^ cells but only after very short treatments. *P = 0.02, **P = 0.01. EIF2α-P levels were normalized by total eIF2α. **B**) Similar to (A), but for cells treated for 24 h. After these long treatments Gz did not inhibit ER stress-induced eIF2α dephosphorylation, it increased CHOP levels. The values in the graphs are averages from 3-4 independent experiments±SE. *P<0.05, **P = 0.002. **C**) Gz showed a minor effect in rescuing ST*Hdh* ^Q111/111^ cells from UPR-induced cell death (Tun for 48 h). ***P =  0.0001.

**Figure 7:**
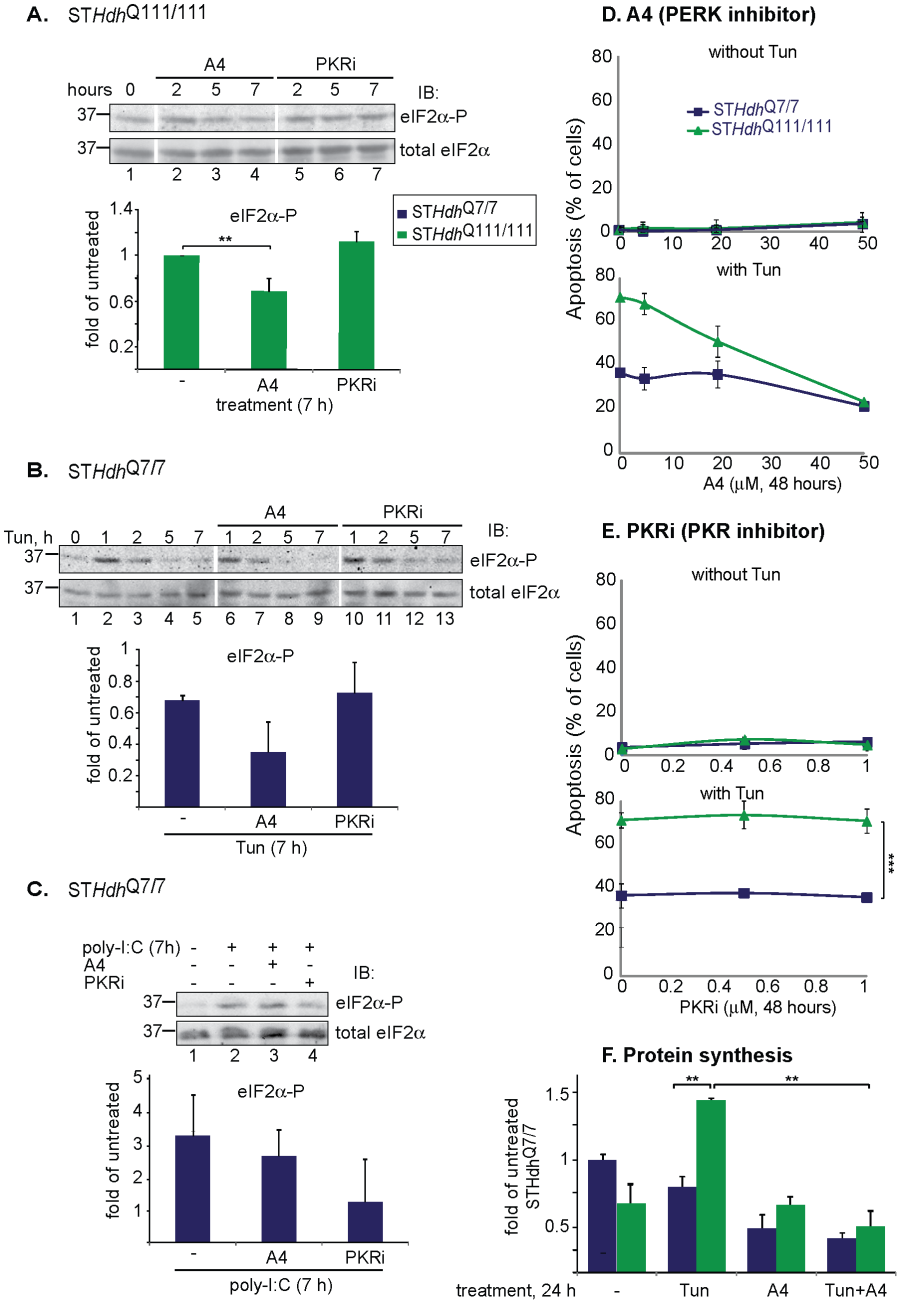
Regulation of phosphorylated eIF2α levels by inhibition of its phosphorylation and rescue of ST*Hdh* ^Q111/111^ cells. **A**) EIF2α phosphorylation in ST*Hdh* ^Q111/111^ cells is PERK-mediated. ST*Hdh* ^Q111/111^ cells left untreated or treated with the PERK inhibitor A4 (50 µM) or the PKR inhibitor PKRi (1 µM) for the indicated times. **P = 0.009. **B**) ER stress-mediated eIF2α phosphorylation is inhibited by A4 and not by PKRi. As in (A), but with ST*Hdh* ^Q7/7^ cells treated for different times with Tun. **C**) PKR-mediated eIF2α phosphorylation is inhibited by PKRi and not by A4. As in (B), but with cells treated for 7h with the PKR inducer poly-I:C (200 µg/ml). **D-E**) A4 rescued ST*Hdh* ^Q111/111^ cells from UPR-induced cell death (Tun for 48 h, D), whereas PKRi had no effect (E). ***P =  0.0001. **F**) Total protein synthesis levels are much increased in ST*Hdh* ^Q111/111^ cells after prolonged ER stress (Tun for 24h) and reduced by A4 (50 µM). **P<0.002 (3 repeat experiments).

We tried to reduce the toxicity caused by Tun with the GADD34-specific inhibitor Gz, shown before to decrease ER stress-induced cytotoxicity in other cells types, apparently by transiently inhibiting eIF2α-P dephosphorylation, delaying the resumption of protein synthesis in stressed cells [25]. As expected, Gz increased eIF2α-P levels (Fig. 6A,B), but it protected only moderately the striatal cells (Fig. 6C, lower panel). Gz slightly delayed eIF2α-P dephosphorylation after short term ER stress in ST*Hdh* ^Q7/7^ cells but not in ST*Hdh* ^Q111/111^ cells (Fig. 6A) and not after prolonged stress (Fig. 6B). Although transient inhibition of eIF2α-P dephosphorylation delays the resumption of protein synthesis in stressed cells, reducing the protein load in the ER, it also induces the expression of pro-apoptotic CHOP. Indeed, Gz increased CHOP expression induced by Tun (Fig. 6B). Gz treatment of cells by itself caused some toxicity (Fig. 6C, upper panel). We reasoned that perhaps the toxicity of Htt111Q is mediated by its effect in increasing the naturally low eIF2α-P that exists in striatal cells. Therefore, we tested whether we could reduce PERK activity and the level of eIF2α phosphorylation, which were elevated by Htt111Q. We tested a compound, A4, that had been shown to inhibit PERK activity *in vitro* [22]. A4 reduced basal eIF2α-P levels in ST*Hdh* ^Q111/111^ cells and inhibited eIF2α phosphorylation in ST*Hdh* ^Q7/7^ cells after short-time induction with Tun (Fig. 7A,B). EIF2α can also be phosphorylated by specific cytosolic kinases not activated by ER stress, among them the double-stranded RNA-activated PKR. We tested an inhibitor of the cytosolic PKR (PKRi), which had no effect on the eIF2α-P level in ST*Hdh* ^Q111/111^ cells, nor on the Tun-induced eIF2α-P, but did inhibit eIF2α phosphorylation cause by the PKR activator poly-I:C (Fig. Fig. 7A-C). These results are consistent with the UPR origin of eIF2α phosphorylation caused by pathogenic Htt. In cells treated with Tun, A4 strongly reduced, in a dose-dependent manner, the additional cytotoxicity in ST*Hdh* ^Q111/111^ cells to the level of ST*Hdh* ^Q7/7^ cells (Fig. 7D). In contrast, PKRi did not affect the cytotoxicity in the striatal cells (Fig. 7E).

We analyzed the levels of total protein synthesis. In ST*Hdh* ^Q111/111^ cells the levels were strongly increased upon prolonged treatment with Tun, compared to the normal levels seen in ST*Hdh* ^Q7/7^ cells (Fig. 7F). This can be explained by the strong induction of GADD34 (Fig. 5) and consequent dephosphorylation of eIF2α, leading to much increased protein synthesis. CHOP induction has also been recently linked to increased protein synthesis [30]. A4 treatment strongly reduced the long-term effect of Tun on protein synthesis in ST*Hdh* ^Q111/111^ cells (Fig. 7F), consistent with its reduction of Tun-induced cytotoxicity in these cells (Fig. 7D). Reduction of eIF2α phosphorylation at initial UPR stages would prevent the later induction of GADD34 and CHOP.

Altogether the results suggest that, for their viability, striatal neurons must keep a very low level of eIF2α phosphorylation, which is altered by ER stress caused by pathogenic huntingtin.

## Discussion

The extremely low basal levels of eIF2α-P in the ST*Hdh* ^Q7/7^ cells compared to other cell types and in the brain striatum compared to other regions would suggest active protein synthesis in striatal neurons, because eIF2α-P inhibits general translation [20]. However, we also found that the total eIF2α levels were low in the striatum. Low eIF2α levels would suggest low translation rates, but this would be compensated by very low eIF2α phosphorylation, remaining in its active state.

Expression of pathogenic Htt increased significantly eIF2α phosphorylation, both in ST*Hdh* ^Q111/111^ cells and in the striatum in mouse brain sections. As the N171-82Q mouse analyzed is a very different Htt model than that from which the ST*Hdh* ^Q111/111^ cells are derived, this suggests generality of the phenomenon caused by polyQ-expanded Htt expression. The higher level of eIF2α-P in ST*Hdh* ^Q111/111^ cells did not protect them, but made them more sensitive to ER stress. Increase in eIF2α-P levels has also been linked recently to cytotoxicity in prion disease [31].

Although inhibition of eIF2α-P dephosphorylation by Gz was shown to be beneficial in some models [25], it was not effective in our study. Transient inhibition of eIF2α-P dephosphorylation might delay the resumption of protein synthesis in stressed cells, reducing the protein load in the ER but it also induces the expression of pro-apoptotic CHOP [32]. It was recently reported that CHOP expression would lead to cell death by increasing the transcription of genes involved in protein synthesis and therefore raising the protein load in a deleterious manner before protein homeostasis is reached. This leads to oxidative stress and apoptosis [30]. Our results are consistent with this model, as ER stress led in the long term to a large increase in protein synthesis in ST*Hdh* ^Q111/111^ cells. The PERK inhibitor restored the lower synthesis levels, as it would reduce CHOP induction. Therefore, there is a delicate balance between the beneficial and harmful effects of eIF2α phosphorylation by PERK. PERK activity was also shown to lead in the long term to apoptosis through additional mechanisms [33]. Consistently, in our study PERK inhibition strongly compensated pathogenic huntingtin toxicity in striatal neurons.

One possible reason for striatal neurons to normally keep a low level of eIF2α-P could be to maintain their functionality for memory and long-term potentiation. These brain activities have been shown to be dependent on a dephosphorylated state of eIF2α, possibly to maintain high translation rates for expression of critical genes [34], [35]. Appearance of ER stress upon expression of polyQ-expanded Htt and the consequent increase in eIF2α-P levels, might explain the early cognitive impairment in HD patients, before motor dysfunction [36]. This should be addressed in future studies. Upon prolonged ER stress, eIF2α-P causes induction of high levels of CHOP in the striatal cells, which would lead to their demise (Fig. 5), consistent with the long-term neurodegeneration starting in the striatum in HD patients [1]. Although many pathways could contribute to pathogenic huntingtin cytotoxicity [4], the fact that PERK inhibition reduces cell death in Htt111Q-expressing cells to the level of WT Htt7Q-expressing cells, implicates this pathway of ER stress as an important factor. The rescue of striatal neurons bearing pathogenic huntingtin by PERK inhibition is encouraging for the development of a promising novel therapy for HD.

## Supporting Information

Figure S1 **Lower initial XBP1s levels in ST** ***Hdh*** **^Q111/111^ cells.** Cells were incubated with Tun (10 µg/ml) for the indicated times and XBP1s mRNA levels were measured by RT-PCR and compared to those of GAPDH. Basal XBP1s levels were very low in ST*Hdh* ^Q111/111^ cells and their upregulation was slower than in the other cell lines. Graph: XBP1s levels, normalized by GAPDH and relative to those in untreated ST*Hdh* ^Q7/7^ cells from 5 experiments ± SE.
(TIF)

Figure S2 **Striatal cells are especially sensitive to prolonged UPR induction or proteasomal inhibition.** This sensitivity is increased by expression of polyQ-expanded Htt. Raw data obtained from FACS analysis of cell cycle progression using propidium iodide (PI). Shown is one representative experiment from those summarized in Fig. 5D. M1 marks fraction of apoptotic cells, beneath G0/G1.
(TIF)
