# Supplementary figures and images for: ER Stress-Induced eIF2-alpha Phosphorylation Underlies Sensitivity of Striatal Neurons to Pathogenic Huntingtin
Source: PLoS One. 2014 Mar 3;9(3):e90803. doi: 10.1371/journal.pone.0090803 (PMC3940916; doi:10.1371/journal.pone.0090803)

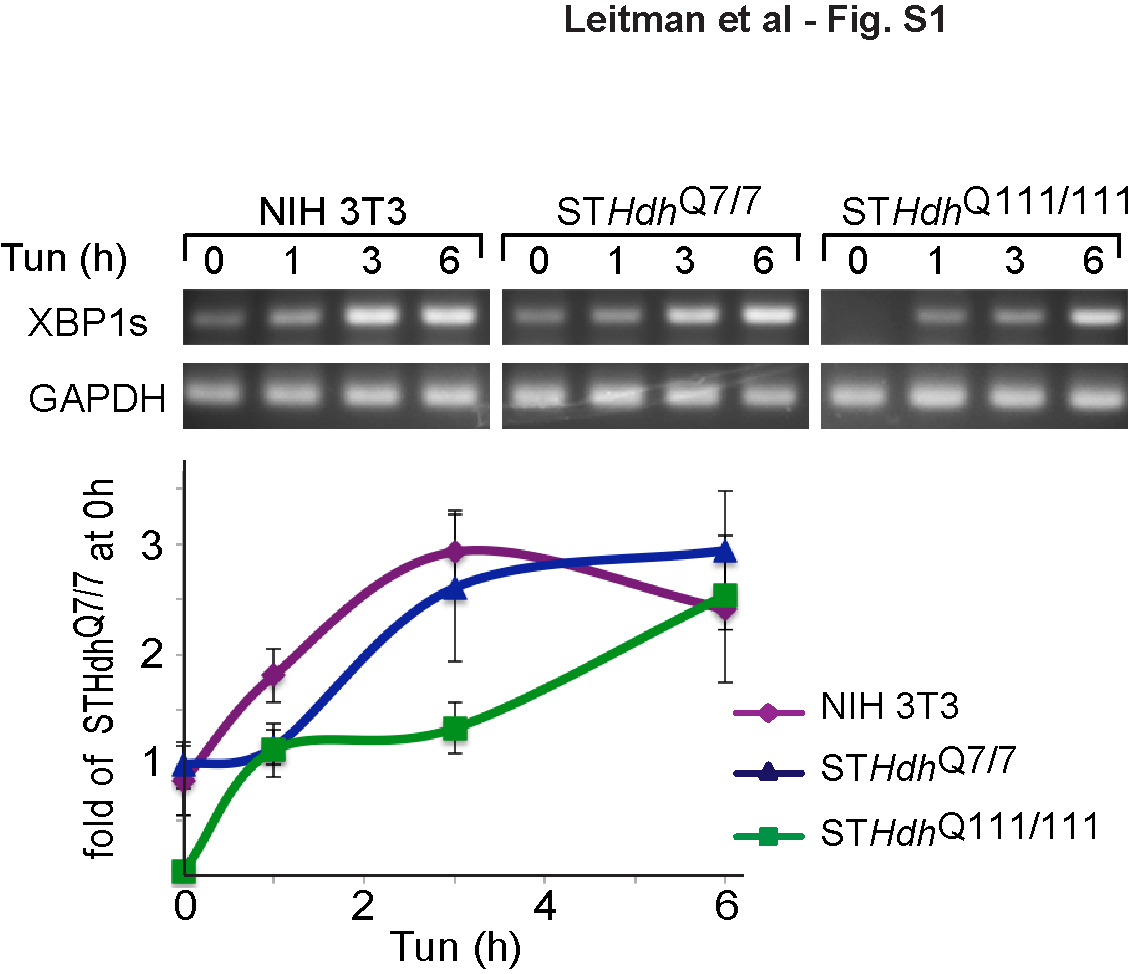

Supplement: Figure S1 — Lower initial XBP1s levels in ST Hdh Q111/111 cells. Cells were incubated with Tun (10 µg/ml) for the indicated times and XBP1s mRNA levels were measured by RT-PCR and compared to those of GAPDH. Basal XBP1s levels were very low in STHdh Q111/111 cells and their upregulation was slower than in the other cell lines. Graph: XBP1s levels, normalized by GAPDH and relative to those in untreated STHdh Q7/7 cells from 5 experiments ± SE. (TIF) [file pone.0090803.s001.tif]

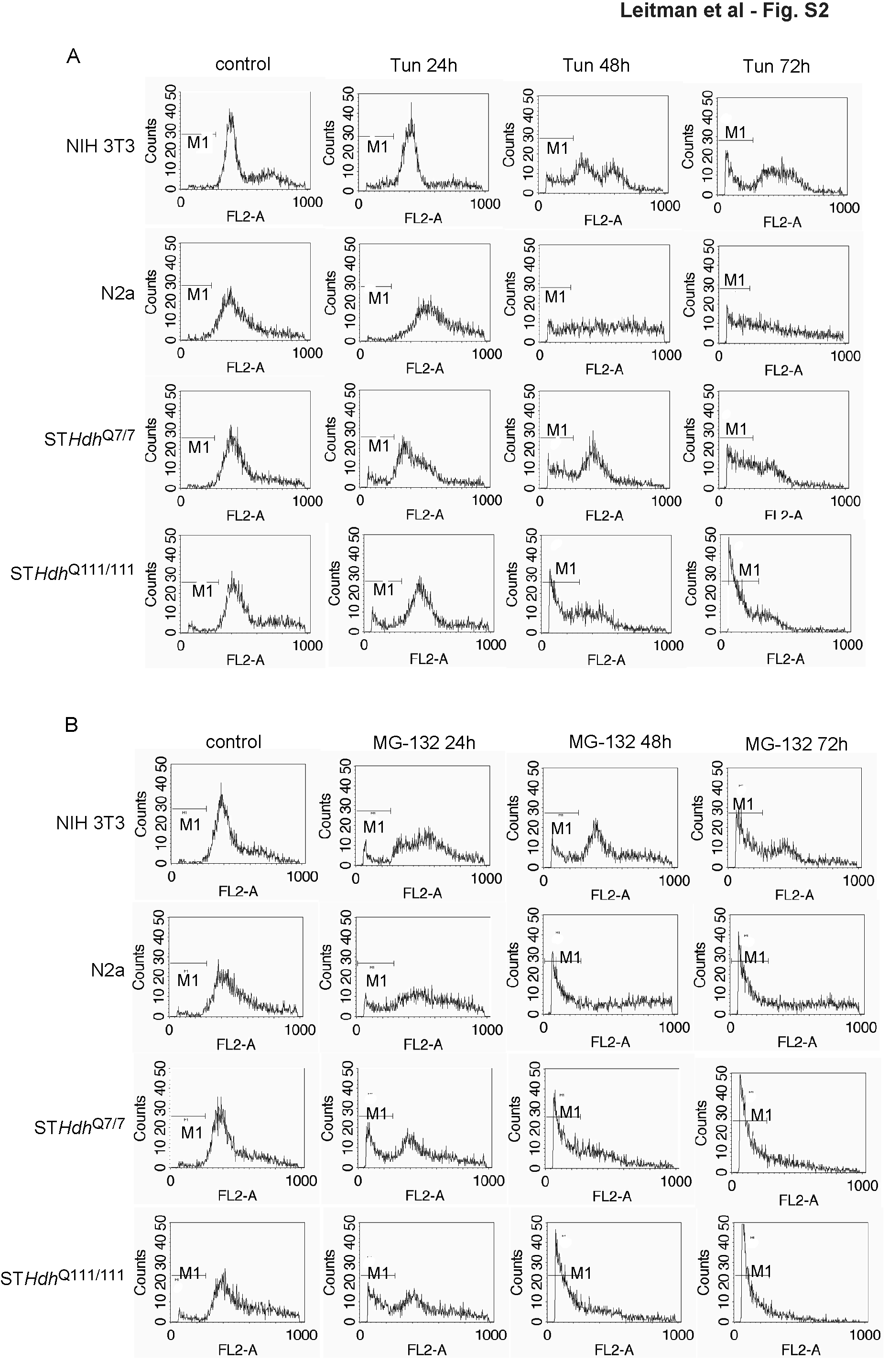

Supplement: Figure S2 — Striatal cells are especially sensitive to prolonged UPR induction or proteasomal inhibition. This sensitivity is increased by expression of polyQ-expanded Htt. Raw data obtained from FACS analysis of cell cycle progression using propidium iodide (PI). Shown is one representative experiment from those summarized in Fig. 5D. M1 marks fraction of apoptotic cells, beneath G0/G1. (TIF) [file pone.0090803.s002.tif]
